# Opposite malaria and pregnancy effect on oral bioavailability of artesunate – a population pharmacokinetic evaluation

**DOI:** 10.1111/bcp.12660

**Published:** 2015-07-22

**Authors:** Frank Kloprogge, Rose McGready, Aung Pyae Phyo, Marcus J Rijken, Warunee Hanpithakpon, Hla Hla Than, Nathar Hlaing, Naw Thida Zin, Nicholas P J Day, Nicholas J White, François Nosten, Joel Tarning

**Affiliations:** 1 Centre for Tropical Medicine and Global Health, Nuffield Department of Medicine, University of OxfordUnited Kingdom; 2 Mahidol-Oxford Tropical Medicine Research Unit, Faculty of Tropical Medicine, Mahidol UniversityBangkok; 3 Shoklo Malaria Research Unit, Mahidol-Oxford Tropical Medicine Research Unit, Faculty of Tropical Medicine, Mahidol UniversityMae Sot, Thailand

**Keywords:** artesunate, dihydroartemisinin, nonmem, population pharmacokinetics, post-partum women, pregnant women

## Abstract

**Aim:**

The aim was to compare the pharmacokinetic properties of artesunate and dihydroartemisinin in the same women: i) pregnant with acute uncomplicated malaria on day 1 and 2, ii) pregnant with convalescent malaria on day 7 and iii) in a healthy state 3 months post-partum on day 1, 2 and 7.

**Methods:**

Non-linear mixed-effects modelling was used to compare plasma concentration–time profiles of artesunate and dihydroartemisinin over 7 days of treatment following oral and intravenous artesunate administration to pregnant women with uncomplicated *Plasmodium falciparum* malaria during their second or third trimesters of pregnancy. The same women were restudied 3 months after delivery when fully recovered. Non-compartmental results of the same study have been published previously.

**Results:**

Twenty pregnant patients on the Thailand-Myanmar border were studied and 15 volunteered to be restudied 3 months post-partum. Malaria and pregnancy had no effect on the pharmacokinetic properties of artesunate or dihydroartemisinin after intravenous artesunate administration. However, malaria and pregnancy had opposite effects on the absorption of orally administered artesunate. Malaria increased the absolute oral bioavailability of artesunate by 87%, presumably by inhibiting first pass effect, whereas pregnancy decreased oral bioavailability by 23%.

**Conclusions:**

The population pharmacokinetic analysis demonstrated opposite effects of malaria and pregnancy on the bioavailability of orally administered artesunate. Lower drug exposures during the second and third trimesters of pregnancy may contribute to lower cure rates and thus the development of drug resistance. Dose optimization studies are required for artesunate containing artemisinin-based combination therapies (ACTs) in later pregnancy.

## What is Already Known about this Subject

- Pregnant patients in their second and third trimesters have lower oral exposures to dihydroartemisinin after oral artesunate dosing which could reduce treatment efficacy and therefore risk the life of the fetus and mother.
- Acute malaria infections increase the oral exposure to dihydroartemisinin.
- The relative contributions of malaria and pregnancy on dihydroartemisinin exposure have not been determined.

## What this Study Adds

- Malaria in pregnancy does not affect the disposition of parenteral artesunate.
- Malaria alters the bioavailability of oral artesunate, resulting in 87% increased systemic drug exposure during the acute phase of malaria.
- Pregnancy alters the bioavailability of oral artesunate, resulting in 23% decreased systemic drug exposure during the second and third trimesters of pregnancy.
- The effects of pregnancy and malaria are independent and concentrations of artesunate and dihydroartemisinin are therefore approximately 25% lower during malaria infection in later pregnancy compared with post-partum patients with a malaria infection.
- An extended treatment or dose increase during pregnancy to achieve equivalent exposures in pregnant women compared with that in non-pregnant women has to be studied further.

## Introduction

Oral artemisinin-based combination therapy (ACT) and parenteral artesunate are the treatments of choice for uncomplicated and severe malaria, respectively 1. Artesunate is a water soluble sodium salt of the hemisuccinate ester of dihydroartemisinin and is commonly co-administered with amodiaquine, mefloquine or sulfadoxine-pyrimethamine for uncomplicated malaria 1.

Artesunate is rapidly converted into its active metabolite, dihydroartemisinin, by hydrolysis at gastric pH, by esterases in the blood, and by cytochrome P450 (CYP) 2A6 2,3. Dihydroartemisinin accounts for most of the antimalarial effect and is glucuronidated by UDP-glucuronosyltransferase (UGT) 1A9 and 2B7 in the gastro-intestinal tract and liver into α-dihydroartemisinin-β-glucuronide and a tetra-hydrofuran of α-dihydroartemisinin-β-glucuronide 4. Malaria alters the pharmacokinetic properties of dihydroartemisinin after oral artesunate administration because the illness reduces the first pass effect resulting in increased systemic exposures during the acute phase of malaria compared with convalescence (>5 days after starting treatment) and healthy volunteers 5–8.

Pregnant women are especially vulnerable to malaria infections 9. Several physiological changes occur during pregnancy, potentially resulting in altered drug pharmacokinetics. For example, decreased gut motility may affect absorption and increased plasma volume, water content and fat content may affect the distribution of the drug 10–12. Furthermore, artesunate and dihydroartemisinin exposures might be decreased during pregnancy due to an increased expression of the cytochrome-P450 isoenzyme (CYP) 2A6 and UDP-glucuronosyltransferase (UGT) 2B7 11,12. In general, artesunate-containing combination therapies show high cure rates (>95%) and are still effective in pregnant women 13–15. However, lower drug exposures in combination with an altered immune system during pregnancy may increase the risk of treatment failures and the development of drug resistance. Indeed, artemether-lumefantrine, another artemisinin derivative, has shown unacceptably low cure rates (<85%) during pregnancy on the Thailand-Myanmar border 9. Pregnant aparasitaemic and asymptomatic women from the Democratic Republic of Congo had an increased dihydroartemisinin apparent elimination clearance (1.39 l kg^–1^ h^–1^) compared with aparasitaemic and asymptomatic post-partum women (1.26 l kg^–1^ h^–1^) and aparasitaemic and asymptomatic non-pregnant women (1.07 l kg^–1^ h^–1^) after a single oral artesunate dose 16. An assessment of these data using a population pharmacokinetic model showed a 42% increase in dihydroartemisinin apparent elimination clearance during pregnancy which suggests that exposure is decreased substantially in pregnant women compared with non-pregnant patients 17.

A non-compartmental analysis has been published previously 8 and showed significant differences after oral administration between pregnant malaria patients and post-partum healthy volunteers. However, a model-independent analysis cannot dissect or quantify the individual contributions of disease and pregnancy to the altered pharmacokinetics. A model-based analysis offers a mechanistic understanding of the antimalarial drug and the influence of physiological processes on the pharmacokinetic properties of the drugs. The aim of this study was to re-evaluate the pharmacokinetic properties of artesunate and dihydroartemisinin in pregnant patients with uncomplicated *Plasmodium falciparum* malaria on the Thailand-Myanmar border and re-assess these post-partum 8 using a simultaneous population pharmacokinetic drug-metabolite modelling approach. To distinguish effects on absorption and disposition, artesunate pharmacokinetics were assessed after both intravenous and oral administration.

## Methods

This study was a re-analysis of a previously published pharmacokinetic study conducted on the North Western border of Thailand and Myanmar in the clinics of the Shoklo Malaria Research Unit from April 2008 until March 2009 8. The model-based analysis in this study can dissect and quantify the malaria and pregnancy effects under the assumption that pregnant malaria patients during their convalescent phase (day 7 during the first visit) have a similar disease state (i.e. healthy) to post-partum healthy volunteers. The pregnant patients were screened weekly for malaria (in an antenatal clinic programme) using microscopy of blood smears. Pregnant women in the second and third trimesters with acute uncomplicated *Plasmodium falciparum* malaria and haematocrit values not lower than 25% were enrolled in the study if they provided written informed consent. Estimated gestational age was measured by dating ultrasound. Post-partum women were followed monthly and the 3 months post-partum visit was postponed in the case of malaria detection or any other illness. Ethical approval for the pharmacokinetic study was obtained from the Faculty of Tropical Medicine, Mahidol University Ethics Committee (TM-IR 029/2005) and the Oxford Tropical Research Ethics Committee (OXTREC 007-05).

Patients were assigned to either group 1 or 2 and all drug intake was completed under supervision. Group 1 received 4 mg kg^–1^ intravenous artesunate on admission followed by 4 mg kg^–1^ oral artesunate for the next 6 days. Group 2 received 4 mg kg^–1^ oral artesunate on admission, 4 mg kg^–1^ intravenous artesunate on day 2 followed by 4 mg kg^–1^ oral artesunate for the next 5 days. The same women were followed-up monthly for 3 months during the post-partum period before receiving the same dose regimen and sampling schedule as during the pregnant period. Intravenous and oral artesunate were manufactured by the Guilin Pharmaceutical Factory, Guangxi in China and repackaged in Thailand by Atlantic Pharmaceuticals. The drugs used for treatment (expiry date: 24 August 2009 and May 2010 for intravenous and oral artesunate) during the pregnancy visit were set aside for their post-partum visit in an air-conditioned pharmacy in Mae Sot.

Venous blood samples (2 ml) were drawn on day 1 and on day 2 at 0, 5, 15, 30, 60, 120, 180, 240 and 360 min or 0, 15, 30, 60, 90, 120, 180, 240 and 360 min after intravenous or oral administration, respectively. Additional venous blood samples were also drawn on day 7 at 0, 60, 120, 240 and 360 min after dose in all patients. Samples were collected in pre-chilled tubes containing sodium fluoride/potassium oxalate as anticoagulant. Samples were inverted five to six times and stored on wet ice until centrifugation at 2000 *g* for 7 min at 4 °C. Plasma samples were stored in liquid nitrogen until transfer to −80 °C for storage. After completion of the study, all samples were transferred on dry ice to the Department of Clinical Pharmacology, Bangkok, Thailand for drug quantification. The laboratory is a participant in the QA/QC proficiency testing programme supported by the Worldwide Antimalarial Resistance Network (WWARN) 18.

Artesunate and dihydroartemisinin were quantified using liquid chromatography-tandem mass-spectrometry 19. The coefficient of variation of the assay was less than 8% at each level of quality control samples and the limit of quantification was set to 1.2 ng ml^–1^ and 2 ng ml^–1^ for artesunate and dihydroartemisinin, respectively.

Plasma concentrations were converted into molar units and modelled simultaneously as their natural logarithms. nonmem 7.2 on a windows 7 operating system was used to perform non-linear mixed-effects modelling. The objective function value (OFV), computed as minus twice the log likelihood of the data, was used to evaluate competing models. A significant improvement between two hierarchical models after inclusion of one additional parameter (one degree of freedom) required at least a 3.84 drop in OFV (*P* = 0.05). Goodness-of-fit diagnostics and physiological plausibility were also considered during the model building process.

A drug-metabolite model was developed to fit artesunate and dihydroartemisinin data simultaneously after oral and intravenous administration under the assumption that artesunate is completely metabolized into dihydroartemisinin 2,3. Different absorption (first order, first order with lag time, transit compartment absorption [*n* = 1–10] and first pass effect of artesunate), distribution (one, two and three compartment distribution), variability (between subject and between dose/visit occasion variability) and residual error (additive, proportional and combined additive and proportional errors on log data) models were considered. Also, a previously described semi-mechanistic liver compartment model was evaluated to describe artesunate and dihydroartemisinin pharmacokinetics simultaneously 20,21. The model building was conducted using the first order conditional estimation method with interaction and the Laplacian estimation method for the M3 method to avoid bias from the large fraction of data below the limit of quantification 22. The initial structural base model was developed based on the intravenous artesunate-dihydroartemisinin data only. Subsequently, rich sampled oral data (days 1 and 2) were evaluated to construct the best performing absorption model.

The best performing structural model was carried forward to covariate model building, where all available oral and intravenous data were used. Bodyweight was tested as an allometric function on the volume of distribution (power fixed to 1), elimination and inter-compartmental clearance parameters (power fixed to either 3/4 or 2/3) to correct for differences in bodyweight between the pregnancy and post-partum visit.

A step-wise covariate model building procedure for all possible covariate-parameter relationships was not practically possible due to the complexity and consequently long run times of the final base model. Therefore, only physiologically plausible covariates were tested on parameters which could affect the systemic exposure of artesunate or dihydroartemisinin.

The disease effect was assessed on bioavailability, first pass effect of artesunate, artesunate elimination clearance and dihydroartemisinin elimination clearance using a variety of descriptors (categorical effect, mildly/moderately unwell or disease count status) during acute malaria on day 1 and day 2 of the study. Patients were considered as having moderately severe disease if plasma creatinine, blood urea nitrogen or liver function tests, including bilirubin (total or direct), aspartate aminotransferase or alanine aminotransferase were raised (25^th^ percentile) or if patients had a fever (>37.5 °C) and tachycardia (>100 beats min^–1^). The patients were classified as having mildly severe disease if none of the above conditions was met. The disease severity count status was calculated as the sum of fulfilled severity criteria used for mildly/moderately unwell allocation.

Pregnancy as a categorical effect, trimester as a categorical effect and estimated gestational age (i.e. 0 weeks for non-pregnant women) as a continuous covariate (linear relationship) were assessed on bioavailability, first pass effect of artesunate, artesunate elimination clearance and dihydroartemisinin elimination clearance.

The robustness of the parameter estimates from the final model was assessed by relative standard errors derived from a bootstrap using 100 resampled datasets. Eta and epsilon shrinkage was calculated to assess the reliability of diagnostic plots, individual parameter estimates and *post hoc* parameter estimates 23. The predictive power of the final model was examined by a visual predictive check derived from 2000 simulations of each individual plasma sample. The visual predictive check represents the 95% confidence intervals of the simulated 5^th^, 50^th^ and 95^th^ percentiles overlaid with the 5^th^, 50^th^ and 95^th^ percentiles of the observed data.

To visualize the impact of the different covariates, simulations (*n* = 500) were performed using parameter estimates from the final population pharmacokinetic model. Three different phases within the study design were evaluated to assess the exposure, a typical pregnant patient with acute *Plasmodium falciparum* malaria (39–64 kg), a typical pregnant woman in the convalescence phase of *Plasmodium falciparum* malaria (39–64 kg) and a typical post-partum healthy volunteer (37–62 kg). Furthermore, a dose-optimization (*n* = 500 simulations) was performed using the final model for oral artesunate (standard treatment for uncomplicated malaria 4.0 mg kg^–1^) in order to evaluate the required dose to achieve similar exposures in pregnant and post-partum patients (40–60 kg) with acute uncomplicated *Plasmodium falciparum* malaria.

## Results

Twenty pregnant women with uncomplicated *Plasmodium falciparum* malaria and 15 women who returned 3 months post-partum as healthy volunteers were studied after receiving artesunate (Table1). The results from the non-compartmental analysis have been presented in full elsewhere 8. Eight of the treated patients remained malaria free until day 63 and 12 had recurrent malaria (7, 1, 1 and 3 of the recurrent malaria cases were caused by *Plasmodium vivax*, *Plasmodium falciparum*, mixed *Plasmodium vivax*/*Plasmodium falciparum* and *Plasmodium falciparum* preceded by *Plasmodium vivax*, respectively). Polymerase chain reaction genotyping showed that two out of the four *Plasmodium falciparum* recurrent infections were novel and two were recrudescent infections. Seventeen women gave birth (mean [range] of 39.2 [35.6–41.5] weeks) to healthy singleton babies and three women were lost to follow-up.

**Table 1:** Demographic summary of the study population

	Pregnant	Post-partum
**Number of patients**	20	15
**Total artesunate dose (mg kg^–1^)**	27.9 (26.8–28.6)	27.4 (4.08–29.0)
**Total number of samples**	920	651
**Sample size (samples/patient)**	46 (46–46)	45 (17–47)
**Admission covariates**		
**Bodyweight (kg)**	48.0 (40.0–64.0)	46.0 (37.0–52.0)
**Mildly/moderately unwell (*n*)**	7/13	-
**Second/third trimester (*n*)**	10/10	-
**Estimated gestational age (weeks)**	25.7 (14.0–38.0)	-

Values are reported as median (range) unless otherwise specified.

**Figure 1:**
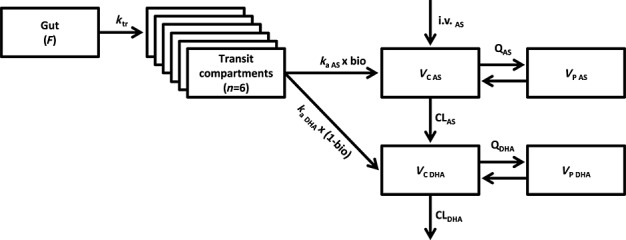
Visual representation of the simultaneous population pharmacokinetic artesunate-dihydroartemisinin model. AS, artesunate; DHA, dihydroartemisinin; *F,* oral bioavailability; *k*_tr_, transit rate constant; *k*_a_, absorption rate constant; bio, fraction of dose absorbed as AS; i.v., intravenous administration; *V*_C_, apparent volume of distribution of central compartment; *V*_P,_ apparent volume of distribution of peripheral compartment; Q, inter-compartmental clearance and CL elimination clearance. *k*_tr_ was calculated as (transit compartments (*n*) +1)/mean transit time

A structural two compartment disposition model for both artesunate and dihydroartemisinin provided a satisfactory fit to the data (Figure1) with no improvement of additional distribution compartments. A non-linear mixed effects model consisting of a flexible transit absorption model (six transit compartments) with the first pass effect of artesunate (i.e. conversion of artesunate into dihydroartemisinin) described the oral absorption phase well and was clearly superior to all other absorption models. Inter-individual variability could be estimated for all fixed effects except for the first pass effect of artesunate, artesunate elimination clearance, central distribution volume of artesunate and peripheral distribution volume of dihydroartemisinin (Table2). Inter-occasion variability could only be estimated for the first pass effect of artesunate (Table2). Residual variability was best described using an additive error model on the log-transformed data (Table2).

**Table 2:** Summary of parameter estimates from the final simultaneous population pharmacokinetic artesunate-dihydroartemisinin model

	Population estimates†	IIV/IOV* [%CV] †
Parameters	(%RSE)‡	(%RSE)‡
***F* (%)**	49.4 (3.53)	27.1 (20.9)
**BIO (%)**	17.1 (6.98)	60.2 (21.3)*
**MTT (h)**	0.407 (7.24)	31.0 (20.2)
**Transit compartments (n)**	6 (*fixed*)	-
**DUR (h)**	0.0167 (*fixed*)	-
***k*_a_ (h^−1^)**	1.57 (8.59)	36.8 (18.9)
***V*_cART_ (l)**	8.80 (5.32)	-
**Q_ART_ (l h^–1^)**	7.51 (12.6)	6.60 (23.2)
***V*_pART_ (l)**	2.43 (13.8)	63.3 (12.8)
**CL_ART_ (l h^–1^)**	170 (6.75)	-
***V*_cDHA_ (l)**	44.3 (6.35)	-
**Q_DHA_ (l h^–1^)**	16.8 (9.7)	-
***V*_pDHA_ (l)**	20.7 (9.35)	-
**CL_DHA_ (l h^–1^)**	60.9 (4.54)	10.4 (22.7)
**Disease effect on *F* (%)**	86.6 (3.50)	-
**Pregnancy effect on *F* (%)**	−23.3 (18.7)	-
**σ_ARS i.v._**	0.856	-
**σ_ARS oral_**	1.16	-
**σ_DHA i.v._**	0.333	-
**σ_DHA oral_**	0.793	-

* inter-occasion variability.

† Population mean values, inter-occasion variability (IOV) and inter-individual variability (IIV) estimated by nonmem. IIV and IOV is presented as 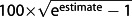. Population parameter estimates are given for a typical non-pregnant patient with a body weight of 46 kg.

‡ The relative standard error (RSE) is calculated as 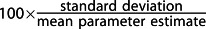 from 99 (out of a total of 100) iterations of a bootstrap.

*F* absolute oral bioavailability of artesunate, BIO first pass metabolism of artesunate, MTT mean transit time, DUR infusion duration, *k*_a_ absorption rate constant, *V*_c_ apparent volume of central compartment, Q inter-compartmental clearance, *V*_p_ apparent volume of peripheral compartment and CL elimination clearance.

Bodyweight was implemented using allometry on all clearance (power fixed to 3/4) and volume parameters (power fixed to 1) and resulted in a minor improvement of the model fit (ΔOFV = −1.49). Disease status was a significant (ΔOFV = −66.4) categorical covariate affecting oral bioavailability (87% higher during the acute phase of malaria). Pregnancy as a categorical covariate on either oral bioavailability (23% lower bioavailability in pregnant patients) or dihydroartemisinin elimination clearance (27% higher in pregnant patients) significantly improved the model fit (ΔOFV = −22.5 and ΔOFV = −45.1, respectively) and was superior compared with trimester and estimated gestational age as covariates, which were unstable and did not significantly improve the model fit. Pregnancy as a categorical covariate on oral bioavailability was carried forward since covariate modelling on intravenous data only could not detect a pregnancy effect on elimination clearance. This was also in agreement with results from the non-compartmental analysis, which could not detect differences in exposure between pregnant and post-partum women after intravenous administration 8.

**Figure 2:**
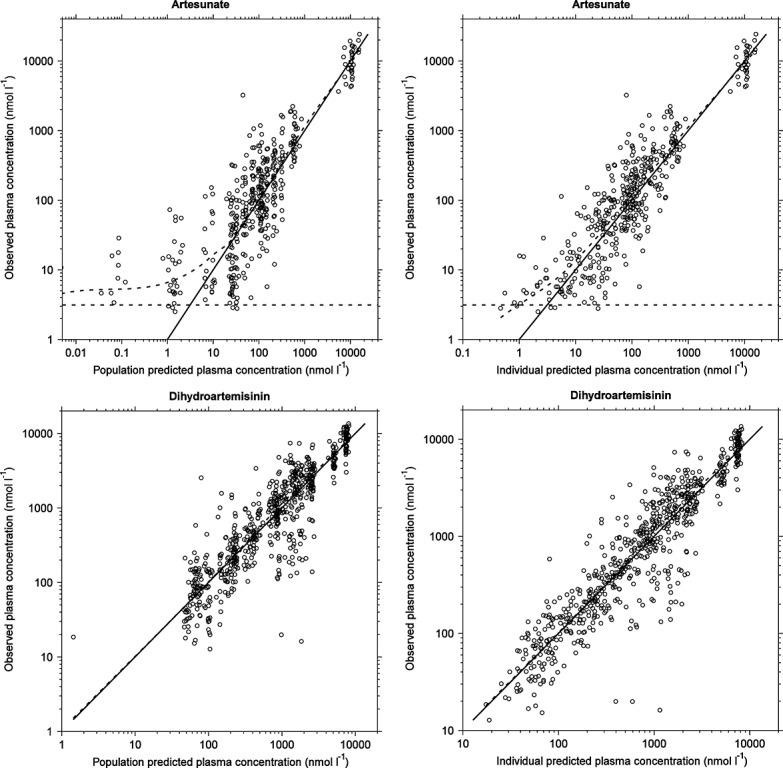
Basic goodness-of-fit diagnostics from the final simultaneous artesunate-dihydroartemisinin model. The line of identity is represented by the black solid line and the trend line (local polynomial regression fitting using 50 evaluations) is represented by the black dashed line

**Figure 3:**
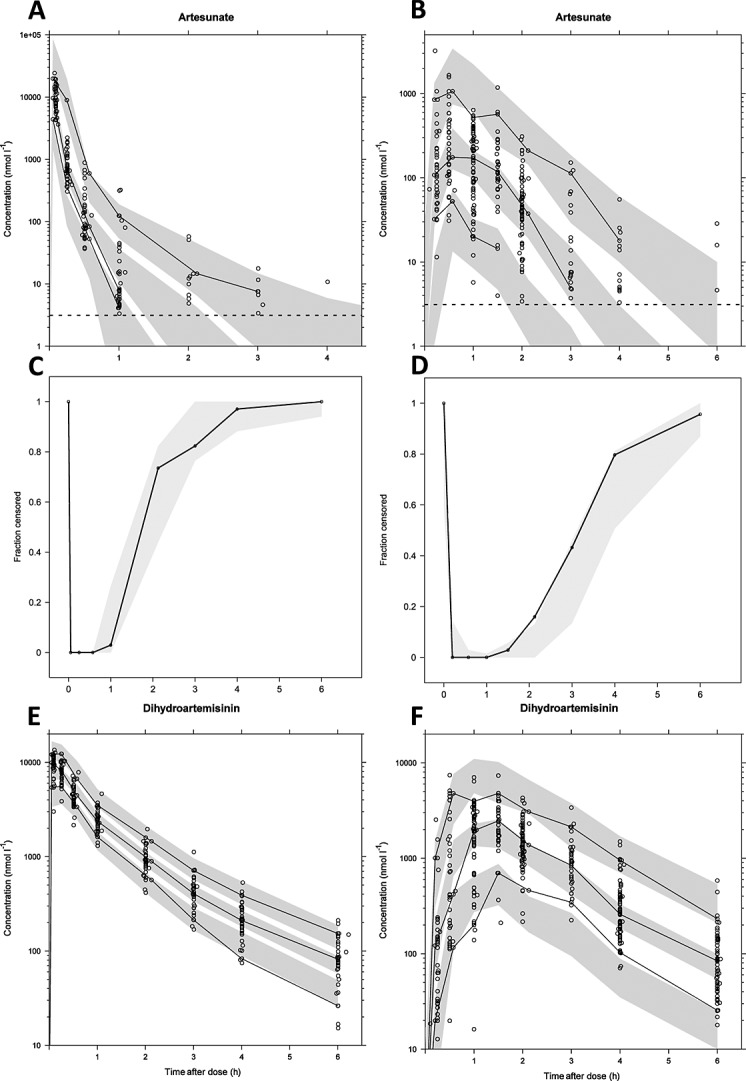
Visual predictive check from the final simultaneous artesunate-dihydroartemisinin model. The figure shows visual predictive checks of artesunate (A) and dihydroartemisinin (E) after intravenous artesunate dosing, and artesunate (B) and dihydroartemisinin (F) after oral artesunate dosing. The black solid lines represent the 5^th^, 50^th^ and 95^th^ percentiles of the observed plasma concentration data and the grey shaded areas represent the confidence intervals of the simulated (*n* = 2000) 5^th^, 50^th^ and 95^th^ percentiles. The observed and simulated fraction of censored artesunate data is shown after intravenous (C) and oral dosing (D). The black solid line represents the observed fraction of plasma samples below the limit of quantification and the grey shaded area represents the confidence interval of the simulated (*n* = 2000) fraction plasma samples below the limit of quantification

Basic goodness-of-fit diagnostics indicated no model misspecification (Figure2) and the prediction-corrected visual predictive check (Figure3) indicated a satisfactory predictive power of the final model. However, the 95^th^ and 5^th^ percentiles of early artesunate concentrations after intravenous and oral artesunate were substantially over and under estimated in the visual predictive check (Figure3A, B). The M3-method 22 was used to avoid bias in parameter estimates as many artesunate data were below the limit of quantification (>45%). This resulted in satisfactory model predictions of the fraction of censored data (Figure3).

Epsilon-shrinkage was below 20% but eta-shrinkages were above 20% for bioavailability, artesunate inter-compartmental clearance and inter-occasion variability on first pass effect of artesunate (shrinkage values between 38.7%–86.6%).

**Figure 4:**
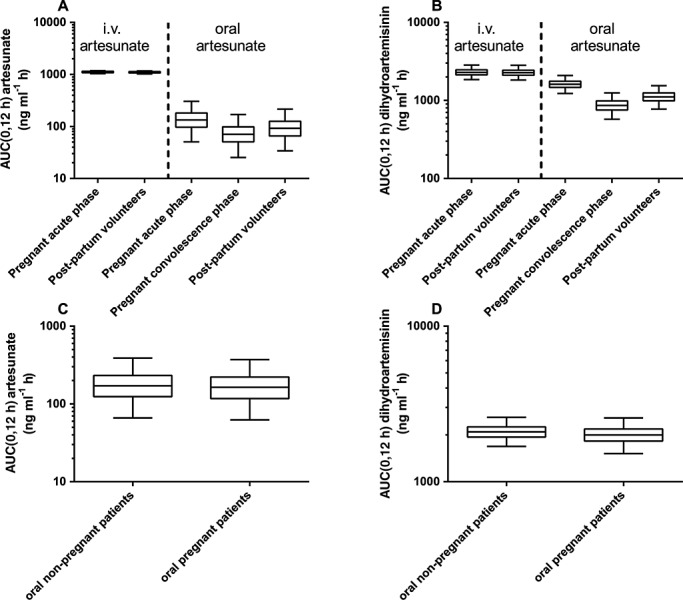
Simulated (*n* = 500) exposures of artesunate and dihydroartemisinin using the final simultaneous population pharmacokinetic artesunate-dihydroartemisinin model. Artesunate (A) and dihydroartemisinin (B) exposures (AUC(0,12 h)) after i.v. (4 mg kg^–1^) and oral (4 mg kg^–1^) dosing during the acute and convalescence malaria phase in pregnant patients (39–64 kg) and during the post-partum visit as healthy volunteers (37–62 kg). Artesunate (C) and dihydroartemisinin (D) exposures (AUC(0,12 h)) after oral (4 mg kg^–1^) dosing in non-pregnant women (40–60 kg) and in pregnant women (40–60 kg) receiving a 25% increased dose. Results are presented using box and whiskers plots (boxes represent 25%–75% and whiskers represent 2.5%–97.5%)

Simulations and *post hoc* estimates, using the final model, showed altered artesunate and dihydroartemisinin exposures as a result of both disease and pregnancy (Figure4 and Table3). Higher oral artesunate and dihydroartemisinin exposures were observed during the acute malaria phase at treatment day 1 and 2 because of the significantly higher bioavailability (*F*) (*post hoc* estimates of *F*_ART_ 12.6% and *F*_DHA_ 71.2%) compared with the convalescence phase at day 7 (*post hoc* estimates of *F*_ART_ 6.19% and *F*_DHA_ 38.4%) and healthy status (*post hoc* estimates of *F*_ART_ 7.62–8.67% and *F*_DHA_ 49.8–49.6%) (Table3). Overall, a 25% lower artesunate and dihydroartemisinin exposure was observed during pregnancy compared with the post-partum period, irrespective of the disease status (i.e. acute malaria, convalescence malaria or healthy status) (Figure4B). Thus, the observed decreased dihydroartemisinin exposure during pregnancy could be corrected by a 25% increase in the administered oral artesunate dose. This would provide equivalent exposures during pregnancy compared with that in post-partum patients with acute uncomplicated *Plasmodium falciparum* malaria (Figure4C, D).

**Table 3:** Summary of *post hoc* parameter estimates from the simultaneous population pharmacokinetic artesunate-dihydroartemisinin model

	Pregnant patients			Post-partum volunteers		
	Acute malaria (days 1 and 2)		Convalescent malaria (day 7)	Days 1 and 2		Day 7
	intravenous	oral	oral	intravenous	oral	oral
	Artesunate					
**AUC(0,12 h) (ng ml^–1^ h)**	1090 (912–1180)	138 (60.3–219)	68.2 (32.8–130)	1090 (1040–1130)	77.5 (56.0–151)	89.6 (51.4–116)
***C*_max_ (ng ml^–1^)**	17 800 (14 800–18 100)	140 (68.4–256)	66.5 (24.9–151)	17 800 (17 600–18 000)	76.1 (42.7–160)	74.1 (40.8–131)
***t*_max_ (h)**	-	1.06 (0.851–1.41)	1.05 (0.860–1.41)	-	1.00 (0.800–1.40)	1.10 (0.800–1.40)
***t*_1/2_ (h)**	0.183 (0.121–0.776)	0.183 (0.121–0.776)	0.183 (0.121–0.776)	0.240 (0.120–0.744)	0.220 (0.120–0.744)	0.240 (0.120–0.744)
**CL (l h^–1^ kg^–1^)**	3.66 (3.40–3.83)	-	-	3.69 (3.58–3.90)	-	-
***V*_d_ (l kg^–1^)**	0.231 (0.217–0.359)	-	-	0.245 (0.217–0.3590	-	-
**CL/*F* (l h^–1^ kg^–1^)**	-	28.9 (18.1–66.3)	59.3 (30.7–124)	-	50.5 (26.9–69.7)	43.3 (33.7–77.9)
***V*_d_/*F* (l kg^–1^)**	-	1.81 (1.35–6.12)	4.29 (2.38–9.12)	-	3.17 (1.66–5.52)	2.85 (2.03–7.74)
***F* (%)**	-	12.6 (5.46–20.6)	6.19 (2.97–12.2)	-	7.62 (5.25–14.0)	8.67 (4.65–10.9)
	Dihydroartemisinin					
**AUC(0,12 h) (ng ml^–1^ h)**	2250 (1860–2850)	1580 (1310–2050)	841 (661–1160)	2240 (1930–2810)	1050 (912–1510)	1040 (912–1510)
***C*_max_ (ng ml^–1^)**	2370 (1980–2420)	779 (484–893)	407 (228–507)	2360 (2300–2450)	549 (322–649)	532 (322–649)
***t*_max_ (h)**	−	1.06 (0.851–1.41)	1.05 (0.860–1.41)	0.100 (0.100–0.200)	1.00 (0.800–1.40)	1.10 (0.800–1.40)
***t*_1/2_ (h)**	1.27 (1.16–1.44)	1.27 (1.16–1.44)	1.27 (1.16–1.44)	1.26 (1.15–1.38)	1.26 (1.15–1.38)	1.26 (1.15–1.38)
**CL (l h^–1^ kg^–1^)**	1.30 (1.04–1.59)	-	-	1.33 (1.07–1.53)	-	-
***V*_d_ (l kg^–1^)**	1.41 (1.41–1.41)	-	-	1.41 (1.41–1.41)	-	-
**CL/*F* (l h^–1^ kg^–1^)**	-	1.89 (1.40–2.23)	3.53 (2.48–4.47)	-	2.71 (2.00–3.31)	2.75 (2.00–3.31)
***V*_d_/*F* (l kg^–1^)**	-	1.99 (1.90–2.28)	3.68 (3.32–4.85)	-	2.84 (2.61–3.56)	2.85 (2.61–3.56)
***F* (%)**	-	71.2 (61.9–74.6)	38.4 (29.1–42.5)	-	49.8 (39.7–54.2)	49.6 (39.7–54.2)

Estimates were calculated as median values (range) from the pharmacokinetic empirical Bayes estimates in pregnant women (40–64 kg) and non-pregnant women (37–52 kg). AUC(0,12 h) area under the plasma concentration curve from 0 to 12 h after the dose, *C*_max_ maximum plasma concentration, *t*_max_ time to reach maximum plasma concentration, *t*_1/2_ elimination half-life, CL elimination clearance after intravenous administration, *V*_d_ apparent volume of distribution after intravenous administration calculated as the sum of central and peripheral apparent volume of distribution and *F* oral bioavailability. After oral administration, elimination clearance and distribution volume are represented as CL/*F* and *V*_d_/*F*.

## Discussion

This study in which each patient was studied twice, first when pregnant with acute (days 1 and 2) or convalescent malaria (day 7) and second when post-partum as healthy volunteers (days 1, 2 and 7) allowed for a quantification of the independent contributions of pregnancy and malaria on the pharmacokinetics of dihydroartemisinin. This assumed that the disease status of pregnant patients on the 7^th^ day of treatment was similar to that in the same women when restudied post-partum as healthy volunteers. Thus, the combination of the study design and the population-based mechanistic analysis methodology enabled the dissection of the disease and pregnancy effects. Artesunate and dihydroartemisinin pharmacokinetics were unaffected by malaria and pregnancy after intravenous artesunate administration which is in agreement with the previous results from a non-compartmental analysis 8. However, malaria and pregnancy showed opposite independent effects on absolute oral bioavailability of artesunate, resulting in 87% increased exposure during the acute disease phase and 23% decreased exposure during pregnancy. The same trend was seen in the previous model-independent analysis but it did not allow for dissecting or quantifying these effects 8.

Disposition pharmacokinetics after intravenous administration were satisfactorily described using two distribution compartments. However, a one compartment distribution model was adequate if only oral artesunate and dihydroartemisinin data were modelled. These findings were in agreement with previous studies and indicate that the distribution phase of artesunate and dihydroartemisinin is attenuated by the absorption phase after oral administration 7,17,24–27. The structural model was further optimized by the implementation of the first pass effect of artesunate into dihydroartemisinin. The slight model misspecification of artesunate in the early absorption and distribution phase (i.e. <30 min) is most likely a result of erratic data. However, the impact of over- and under-predictions of early artesunate concentrations is not expected to have clinical implications as dihydroartemisinin is responsible for the major antimalarial activity, and it should therefore not have any impact on the onset of the antimalarial effect. The first pass effect of artesunate can be explained physiologically by hydrolysis at gastric pH, esterases in the blood and hepatic CYP2A6 activity. A semi-physiological liver compartment model 20,21 was also assessed in the model building process but did not converge successfully due to increased complexity and a relatively small sample size.

A relatively increased exposure of artesunate and dihydroartemisinin during the acute malaria phase on day 1 and 2 (87% increase in oral bioavailability) compared with the convalescence phase (at day 7) and post-partum volunteers could be explained by a reduced first pass effect of artesunate. The unbalanced sampling schedule (days 1 and 2 dense sampling and day 7 sparse sampling), might bias the results but this is unlikely to result in falsely detected disease effects as the finding is in agreement with previous studies 5–7. Artesunate is converted, pre-systemically and systemically, into dihydroartemisinin by hydrolysis at gastric pH, by esterases in the blood and by hepatic CYP 2A6, and dihydroartemisinin is glucuronidated pre-systemically and in the liver. The relatively increased exposure of both artesunate and dihydroartemisinin during acute malaria suggest that one or several of these pathways have reduced activity during acute malaria. However, it remains possible that the absorption of artesunate is also increased in patients due to altered gastrointestinal tract conditions and motility.

The bioavailability of artesunate decreased by 23% during pregnancy compared with the post-partum phase 3 months later (after compensating for the disease effect). Pregnancy had a statistically greater impact on the elimination clearance of dihydroartemisinin compared with the bioavailability of artesunate, but the effect size was similar (27% *vs*. 23%) irrespectively of parameterization. Both the non-compartmental and model-based analysis of intravenous data showed no pregnancy effect on the elimination clearance of artesunate or dihydroartemisinin. Artesunate and dihydroartemisinin are highly-extracted drugs (hepatic extraction ratio > 0.7). Under the assumption of the well-stirred model, hepatic elimination clearance, and thus intravenous exposure, is therefore not affected to a large degree by changes in intrinsic clearance. However, the first pass effect is proportional to the intrinsic clearance for high-extracted drugs. Pregnancy was therefore retained as a categorical covariate on the absolute bioavailability of oral artesunate in the final model. Estimated gestational age as a continuous covariate was not superior to pregnancy as a categorical covariate which reflects the relatively small sample size (i.e. 10 patients in the second and 10 patients in the third trimester). An additional benefit of implementing pregnancy as a categorical covariate (instead of estimated gestational age as a continuous covariate) is the simplified dose adjustments needed for pregnant women with malaria.

The decreased bioavailability in pregnant women is in agreement with previous findings where dihydroartemisinin exposure decreased by 42% in pregnant women compared with a non-pregnant control group 17. Similarly, the estimated exposure decreased by 38% in pregnant women compared with a non-pregnant control group after oral dihydroartemisinin treatment 28. This pregnancy effect was also confirmed by a literature comparison as oral artesunate and dihydroartemisinin exposures during acute malaria in this study (138 [60.3–219] ng ml^–1^ h and 1580 [1310–2050] ng ml^–1^ h) were substantially lower compared with non-pregnant patients with acute uncomplicated malaria (279–386 ng ml^–1^ h and 3854–4680 ng ml^–1^ h) 29,30. However, ratios from this literature comparison should be interpreted with caution as other non-pregnancy related factors (e.g. parasitaemia, ethnic differences and sample handling) may also have contributed to the differences in exposure. The decreased exposure is most likely a result of increased pre-systemic CYP 2D6 and UGT 2B7 activity during pregnancy 11,12. The reduced antimalarial exposure during pregnancy might result in an increased risk of treatment failure and could therefore accelerate the development of resistance.

The magnitude of the pregnancy effect found in this study (23% lower exposure) is comparable but slightly lower than other studies (37.5% – ≥ 50% lower exposure) 17,28–30. This might be explained by the fact that the control group in this study were post-partum women and post-partum women (3 months) have been reported to have a higher apparent dihydroartemisinin elimination clearance (1.26 [0.63–2.32] l kg^–1^ h^–1^) compared with non-pregnant women (1.07 [0.53–1.89] l kg^–1^ h^–1^) 16. This implies that dose optimizations for pregnant women derived from the developed model in the current study might still under-estimate the doses required to reach sufficient dihydroartemisinin exposures in pregnant patients. Exposures during the convalescence phase might also remain slightly increased if there was an extended malaria disease effect and this would contribute to the relatively small pregnancy effect seen in this study. Dose optimizations described in this study should therefore be interpreted as pilot results for further research. A meta-analysis with pregnant patients, post-partum patients and non-pregnant patients is therefore needed to confirm the current findings (i.e. trend of lower exposure during pregnancy) and derive appropriate dosing.

The *in silico* dose comparison performed in the current analysis which simulated pregnant and post-partum patients would not have been possible with a non-compartmental analysis. The comparison of exposure was focused on dihydroartemisinin pharmacokinetics as artesunate was considered a pro-drug, contributing only a small proportion of the overall antimalarial activity. Variability in the simulated exposures may be underestimated due to the high eta-shrinkage on certain parameters (38.7%–86.6%) although the medians should remain accurate. High eta-shrinkage might result in an over-prediction of the fraction of pregnant patients displaying an exposure above the lower limit of the therapeutic window. Similarly it may result in under-prediction of toxic side effects. However, adverse events with artesunate-dihydroartemisinin are not expected since doses up to 6 mg kg^–1^ (which would mean a 50% dose increase) have proved to be safe in patients 29. Furthermore, since the reference population, post-partum patients in the current simulations also suffer from eta-shrinkage relative comparisons between pregnant and post-partum patients remain valid. A 25% higher dose in pregnant patients would result in an equivalent exposure to dihydroartemisinin compared with that in post-partum patients. A larger dose increase would be needed to provide exposures similar to those in non-pregnant patients with acute malaria. This would reduce the risk of treatment failures in pregnancy and therefore decrease the risk of the development and spread of artemisinin resistance. However, information regarding toxicity for the foetus at higher dose levels is limited. Furthermore, a dose increase is not straightforward as ACTs are commonly fixed formulations and the partner drug might be unaffected by pregnancy. An extended treatment regimen (e.g. standard dose over 4 or 5 days) might be an alternative dose optimization strategy in those cases and it would result in an increased exposure time and be more beneficial from a toxicological perspective compared with a dose increase. Prospective dose optimization studies are needed with current ACTs to assess pharmacokinetics, clinical impact, safety and the practical implementation of an increased dose.

In conclusion, malaria (87% increase) and pregnancy (23% decrease) showed opposite independent effects on the absolute oral bioavailability of artesunate. Further dose optimization studies are needed in this vulnerable group to achieve equivalent oral exposures in pregnant and non-pregnant patients with malaria and thereby minimize the risk of treatment failures and the development of drug resistance.

## Competing Interests

The Wellcome Trust is a UK-based medical research charity and is independent of all drug companies.

It has no financial links with the manufacturers of either the diagnostic tests or the drugs used in this study. The other authors declare no conflict of interest.

We sincerely thank the pregnant women for their co-operation in completing this study. We thank also the medical, nursing, midwifery, laboratory, logistic and administrative staff from SMRU and Mahidol Oxford Research Unit (MORU) in Bangkok. The drug assays were supported by the Malaria in Pregnancy consortium, which is funded through a grant from the Bill and Melinda Gates Foundation to the Liverpool School of Tropical Medicine. This investigation was part of the Wellcome Trust Mahidol University-Oxford Tropical Medicine Research Programme supported by the Wellcome Trust of Great Britain.

## Contributors

RM, AP, MR, HT, NH, NZ, ND, NW, JT and FN planned and conducted the clinical study. WH performed the drug analysis and FK and JT conducted the pharmacokinetic analysis. FK and JT drafted the manuscript. All authors reviewed the manuscript critically for important intellectual content and approved the final version.

## References

[b1] World Health Organization (2010). Guidelines for the treatment of malaria.

[b2] Li XQ, Bjorkman A, Andersson TB, Gustafsson LL, Masimirembwa CM (2003). Identification of human cytochrome P(450)s that metabolise anti-parasitic drugs and predictions of in vivo drug hepatic clearance from *in vitro* data. Eur J Clin Pharmacol.

[b3] Navaratnam V, Mansor SM, Sit NW, Grace J, Li Q, Olliaro P (2000). Pharmacokinetics of artemisinin-type compounds. Clin Pharmacokinet.

[b4] Ilett KF, Ethell BT, Maggs JL, Davis TM, Batty KT, Burchell B, Binh TQ, Thu le TA, Hung NC, Pirmohamed M, Park BK, Edwards G (2002). Glucuronidation of dihydroartemisinin *in vivo* and by human liver microsomes and expressed UDP-glucuronosyltransferases. Drug Metab Dispos.

[b5] Teja-Isavadharm P, Watt G, Eamsila C, Jongsakul K, Li Q, Keeratithakul G, Sirisopana N, Luesutthiviboon L, Brewer TG, Kyle DE (2001). Comparative pharmacokinetics and effect kinetics of orally administered artesunate in healthy volunteers and patients with uncomplicated falciparum malaria. Am J Trop Med Hyg.

[b6] Binh TQ, Ilett KF, Batty KT, Davis TM, Hung NC, Powell SM, Thu LT, Thien HV, Phuong HL, Phuong VD (2001). Oral bioavailability of dihydroartemisinin in Vietnamese volunteers and in patients with falciparum malaria. Br J Clin Pharmacol.

[b7] Newton P, Suputtamongkol Y, Teja-Isavadharm P, Pukrittayakamee S, Navaratnam V, Bates I, White N (2000). Antimalarial bioavailability and disposition of artesunate in acute falciparum malaria. Antimicrob Agents Chemother.

[b8] McGready R, Phyo AP, Rijken MJ, Tarning J, Lindegardh N, Hanpithakpon W, Than HH, Hlaing N, Zin NT, Singhasivanon P, White NJ, Nosten F (2012). Artesunate/dihydroartemisinin pharmacokinetics in acute falciparum malaria in pregnancy: absorption, bioavailability, disposition and disease effects. Br J Clin Pharmacol.

[b9] McGready R, Tan SO, Ashley EA, Pimanpanarak M, Viladpai-Nguen J, Phaiphun L, Wustefeld K, Barends M, Laochan N, Keereecharoen L, Lindegardh N, Singhasivanon P, White NJ, Nosten F (2008). A randomised controlled trial of artemether-lumefantrine versus artesunate for uncomplicated plasmodium falciparum treatment in pregnancy. PLoS Med.

[b10] Dawes M, Chowienczyk PJ (2001). Drugs in pregnancy. Pharmacokinetics in pregnancy. Best Pract Res Clin Obstet Gynaecol.

[b11] Anderson GD (2005). Pregnancy-induced changes in pharmacokinetics: a mechanistic-based approach. Clin Pharmacokinet.

[b12] Anderson GD (2006). Using pharmacokinetics to predict the effects of pregnancy and maternal-infant transfer of drugs during lactation. Expert Opin Drug Metab Toxicol.

[b13] Tangpukdee N, Krudsood S, Thanachartwet V, Pengruksa C, Phophak N, Kano S, Li G, Brittenham GM, Looareesuwan S, Wilairatana P (2008). Efficacy of Artequick versus artesunate-mefloquine in the treatment of acute uncomplicated falciparum malaria in Thailand. Southeast Asian J Trop Med Public Health.

[b14] Tangpukdee N, Krudsood S, Thanachartwet W, Chalermrut K, Pengruksa C, Srivilairit S, Silachamroon U, Wilairatana P, Phongtananant S, Kano S, Looareesuwan S (2005). An open randomized clinical trial of Artekin *vs* artesunate-mefloquine in the treatment of acute uncomplicated falciparum malaria. Southeast Asian J Trop Med Public Health.

[b15] van Vugt M, Brockman A, Gemperli B, Luxemburger C, Gathmann I, Royce C, Slight T, Looareesuwan S, White NJ, Nosten F (1998). Randomized comparison of artemether-benflumetol and artesunate-mefloquine in treatment of multidrug-resistant falciparum malaria. Antimicrob Agents Chemother.

[b16] Onyamboko MA, Meshnick SR, Fleckenstein L, Koch MA, Atibu J, Lokomba V, Douoguih M, Hemingway-Foday J, Wesche D, Ryder RW, Bose C, Wright LL, Tshefu AK, Capparelli EV (2011). Pharmacokinetics and pharmacodynamics of artesunate and dihydroartemisinin following oral treatment in pregnant women with asymptomatic Plasmodium falciparum infections in Kinshasa DRC. Malar J.

[b17] Morris CA, Onyamboko MA, Capparelli E, Koch MA, Atibu J, Lokomba V, Douoguih M, Hemingway-Foday J, Wesche D, Ryder RW, Bose C, Wright L, Tshefu AK, Meshnick S, Fleckenstein L (2011). Population pharmacokinetics of artesunate and dihydroartemisinin in pregnant and non-pregnant women with malaria. Malar J.

[b18] Lourens C, Lindegardh N, Barnes KI, Guerin PJ, Sibley CH, White NJ, Tarning J (2014). Benefits of a pharmacology antimalarial reference standard and proficiency testing program provided by the Worldwide Antimalarial Resistance Network (WWARN). Antimicrob Agents Chemother.

[b19] Hanpithakpong W, Kamanikom B, Dondorp AM, Singhasivanon P, White NJ, Day NP, Lindegardh N (2008). A liquid chromatographic-tandem mass spectrometric method for determination of artesunate and its metabolite dihydroartemisinin in human plasma. J Chromatogr.

[b20] Gordi T, Xie R, Huong NV, Huong DX, Karlsson MO, Ashton M (2005a). A semiphysiological pharmacokinetic model for artemisinin in healthy subjects incorporating autoinduction of metabolism and saturable first-pass hepatic extraction. Br J Clin Pharmacol.

[b21] Gordi T, Xie R, Jusko WJ (2005b). Semi-mechanistic pharmacokinetic/pharmacodynamic modelling of the antimalarial effect of artemisinin. Br J Clin Pharmacol.

[b22] Beal SL (2001). Ways to fit a PK model with some data below the quantification limit. J Pharmacokinet Pharmacodyn.

[b23] Savic RM, Karlsson MO (2009). Importance of shrinkage in empirical bayes estimates for diagnostics: problems and solutions. AAPS J.

[b24] Li Q, Cantilena LR, Leary KJ, Saviolakis GA, Miller RS, Melendez V, Weina PJ (2009). Pharmacokinetic profiles of artesunate after single intravenous doses at 0.5, 1, 2, 4, and 8 mg/kg in healthy volunteers: a phase I study. Am J Trop Med Hyg.

[b25] Tan B, Naik H, Jang IJ, Yu KS, Kirsch LE, Shin CS, Craft JC, Fleckenstein L (2009). Population pharmacokinetics of artesunate and dihydroartemisinin following single- and multiple-dosing of oral artesunate in healthy subjects. Malar J.

[b26] Simpson JA, Agbenyega T, Barnes KI, Di Perri G, Folb P, Gomes M, Krishna S, Krudsood S, Looareesuwan S, Mansor S, McIlleron H, Miller R, Molyneux M, Mwenechanya J, Navaratnam V, Nosten F, Olliaro P, Pang L, Ribeiro I, Tembo M, van Vugt M, Ward S, Weerasuriya K, Win K, White NJ (2006). Population pharmacokinetics of artesunate and dihydroartemisinin following intra-rectal dosing of artesunate in malaria patients. PLoS Med.

[b27] Karunajeewa HA, Ilett KF, Dufall K, Kemiki A, Bockarie M, Alpers MP, Barrett PH, Vicini P, Davis TM (2004). Disposition of artesunate and dihydroartemisinin after administration of artesunate suppositories in children from Papua New Guinea with uncomplicated malaria. Antimicrob Agents Chemother.

[b28] Tarning J, Rijken MJ, McGready R, Phyo AP, Hanpithakpong W, Day NP, White NJ, Nosten F, Lindegardh N (2012). Population pharmacokinetics of dihydroartemisinin and piperaquine in pregnant and nonpregnant women with uncomplicated malaria. Antimicrob Agents Chemother.

[b29] Das D, Tripura R, Phyo AP, Lwin KM, Tarning J, Lee SJ, Hanpithakpong W, Stepniewska K, Menard D, Ringwald P, Silamut K, Imwong M, Chotivanich K, Yi P, Day NP, Lindegardh N, Socheat D, Nguon C, White NJ, Nosten F, Dondorp AM (2013). Effect of high-dose or split-dose artesunate on parasite clearance in artemisinin-resistant falciparum malaria. Clin Infect Dis.

[b30] Saunders D, Khemawoot P, Vanachayangkul P, Siripokasupkul R, Bethell D, Tyner S, Se Y, Rutvisuttinunt W, Sriwichai S, Chanthap L, Lin J, Timmermans A, Socheat D, Ringwald P, Noedl H, Smith B, Fukuda M, Teja-Isavadharm P (2012). Pharmacokinetics and pharmacodynamics of oral artesunate monotherapy in patients with uncomplicated Plasmodium falciparum malaria in western Cambodia. Antimicrob Agents Chemother.

